# Dietary Intervention with β-Lactoglobulin-Derived Peptides and a Specific Mixture of Fructo-Oligosaccharides and *Bifidobacterium breve* M-16V Facilitates the Prevention of Whey-Induced Allergy in Mice by Supporting a Tolerance-Prone Immune Environment

**DOI:** 10.3389/fimmu.2017.01303

**Published:** 2017-10-23

**Authors:** Atanaska I. Kostadinova, Alba Pablos-Tanarro, Mara A. P. Diks, Betty C. A. M. van Esch, Johan Garssen, Léon M. J. Knippels, Linette E. M. Willemsen

**Affiliations:** ^1^Division of Pharmacology, Utrecht Institute for Pharmaceutical Sciences, Utrecht University, Utrecht, Netherlands; ^2^Department of Immunology, Nutricia Research, Utrecht, Netherlands; ^3^Instituto de Investigación en Ciencias de la Alimentación (CIAL, CSIC-UAM), Madrid, Spain

**Keywords:** β-lactoglobulin, cow’s milk allergy, dendritic cells, oral tolerance, peptides, regulatory T cells, short-chain fatty acids, synbiotics

## Abstract

Cow’s milk allergy (CMA) prevails in infants and brings increased risk of developing other allergic diseases. Oral administration of specific β-lactoglobulin (BLG)-derived peptides (PepMix) and a specific blend of short- and long-chain fructo-oligosaccharides and *Bifidobacterium breve* M-16V (FF/*Bb*) was found to partially prevent CMA development in mice. In this study, we aimed to expand the knowledge on the preventive potential and the underlying mechanisms of this approach. Three-week-old female C3H/HeOuJ mice were orally exposed to PepMix±FF/*Bb* prior to a 5-week oral sensitization with whole whey and cholera toxin as an adjuvant. The acute allergic skin response was determined after an intradermal challenge with whole whey protein. Following an oral challenge with whey, regulatory T cells (T_regs_) in the small intestine lamina propria (SI-LP) and mRNA expression of immune markers in the Peyer’s patches (PP) were investigated. The early impact of PepMix and FF/*Bb* interventions on the immune system during the oral tolerance (OT) induction phase was investigated after the last OT administration. Pre-exposing mice to PepMix+FF/*Bb* partially prevented the acute allergic skin response compared to PBS and increased T_regs_ and activated T cells in the SI-LP compared to sham-sensitized mice. It also increased the mRNA expression of Tbet over GATA3 in the PP of whey-sensitized mice. Directly upon the 6-day OT phase, FF/*Bb* intervention enhanced cecal content levels of propionic and butyric acid in PepMix-fed mice and the former was positively correlated with Foxp3^+^ cell numbers in the colon. In the PP of PepMix+FF/*Bb*-exposed mice, IL-22 mRNA expression increased and IL-10 followed the same tendency, while the Foxp3 expression was increased over GATA3 and RorγT. In the colon, the Tbet mRNA expression increased over GATA3, while IL-22 decreased. In addition, the Foxp3^+^/GATA3^+^ and regulatory/effector T cell ratios in the mesenteric lymph nodes and the CD11b^+^/CD11b^−^ conventional dendritic cells ratio in the SI-LP were increased. In conclusion, the FF/*Bb* diet facilitates the capacity of the specific BLG-peptides to partially prevent the allergic response after sensitization to whole whey protein, possibly by creating a tolerance-prone environment during the OT phase. Such a dietary intervention might contribute to tailoring successful strategies for CMA prevention.

## Introduction

IgE-mediated food allergy is the most prominent cause of early life anaphylaxis (1). Even though associated with a high rate of natural tolerance and outgrow within the first 5 years of life, cow’s milk allergy (CMA) is the earliest food allergy in infancy and affects 2–3% of children (2). Both casein and whey proteins from cow’s milk can trigger CMA. In the whey fraction, β-lactoglobulin (BLG) is one of the main allergens. In an increasing number of patients, CMA fails to resolve spontaneously and persists for life which is often associated with a more severe phenotype or increased risk for other allergic disorders (3). Although allergen-specific immunotherapy is being developed and can lead to desensitization of patients while on therapy, CMA management in the clinic mainly involves the avoidance of the symptom-eliciting food (4). This may affect children’s growth and impair the quality of life of patients and their families (5).

For many years, oral tolerance (OT) has been the focus of research, as it might provide the tool for developing successful preventive as well as therapeutic strategies for CMA. OT is the phenomenon of developing a systemic immune hyporesponsiveness to harmless food proteins encountered *via* the oral route (6, 7). The mechanisms underlying OT induction remain to be elucidated, but current knowledge suggests it is an active multiple-step process. It involves the uptake of a food antigen, its presentation by antigen-presenting cells (APC), and the induction of anergy, deletion, and/or regulatory T cell (T_regs_) responses in the secondary lymphoid tissues, such as the mesenteric lymph nodes (MLN) (8). According to literature, the primary cellular mechanism to be induced depends on the amount of antigen ingested, where high-dose antigen results in anergy/deletion and low-dose antigen leads to active induction of T_regs_ (9). In the intestinal mucosa, dendritic cells (DC) are considered crucial mediators driving the T cell polarization toward a regulatory or effector phenotype (10). CD103^+^ DC play an important role in OT development by inducing allergen-specific T_regs_ *via* a TGF-β and retinoic acid-dependent mechanism (8, 11–14). From all different T cell subsets, Foxp3^+^ T_regs_ have mostly been researched in the context of OT. Their central and suppressive role in allergy is further supported by research in patients with a non-coding deletion of the *FOXP3* gene who have defective T_regs_ frequency and functionality and exhibit a severe food allergic phenotype, especially against cow’s milk proteins (CMP) (15).

In the past years, avoidance of the culprit food has been the gold standard for primary CMA prevention. However, this paradigm was challenged as allergen avoidance in early life was associated with increased risk of sensitization (16). OT is a strictly antigen-dependent process (17) and oral exposure to antigens in early life might be essential for successful OT development (18). Therefore, when avoiding the allergen, the normal acquisition of OT is evaded while alternative routes of allergen exposure, such as *via* the skin, may increase the risk of sensitization (19, 20). More recently, clinical trials on the early introduction of whole allergen have shown efficacy in reducing the prevalence of peanut allergy (21) while this approach has been less successful for egg (22, 23). Furthermore, the use of intact milk proteins in formulas is not recommended in the first 4 months of life for infants who are genetically predisposed to allergy and are not exclusively breast-fed (24), due to the risk of triggering oral sensitization or allergic symptoms (25, 26). For this reason, current recommendations for primary prevention in high-risk infants who are not exclusively breast-fed include the use of CMP-based partial or extensive hydrolyzed protein formulas (24, 25, 27–32). However, a recent systematic review reported lack of consistent evidence to support the suggestion that partially and extensively hydrolyzed formulas have a protective role and can reduce the risk of allergic disease in humans (33). This further encourages the search for alternative, safer approaches for inducing OT in at-risk infants.

Specific, T cell-epitope-containing peptides (34–38) can alternatively be utilized to avoid potential sensitization or allergic symptoms in high-risk infants. Preclinical studies in rodents have demonstrated that the sensitizing capacity of BLG can be reduced when mice are co-immunized with intact BLG and BLG-derived peptides (39). Careful selection of specific peptides after hydrolysis and their enrichment in a hydrolyzed protein formula has shown reduction of the allergic response to the naïve protein in a murine CMA model (37). Furthermore, a mixture of few synthetically prepared BLG-peptides has been reported to reduce the allergic skin response in a mouse model of CMA (36).

An adjunctive strategy for early life allergy prevention through dietary interventions is the use of dietary supplements with immunomodulatory properties, such as synbiotics. Such components might contribute by actively improving maturation of the mucosal immune system and facilitating OT induction. Combining non-digestible oligosaccharides, such as short- and long-chain fructo-oligosaccharides (scFOS/lcFOS; FF) with a probiotic strain with an anti-allergic activity, such as *Bifidobacterium breve* M-16V (*Bb*) into a synbiotic diet (FF/*Bb*), has previously been found to effectively prevent house dust mite-induced allergic asthma in mice (40) as well as to increase the percentage of beneficial bifidobacteria in feces of cow’s milk allergic infants (41). Therefore, an adjunctive dietary intervention with synbiotics is of interest for developing promising strategies for CMA prevention.

In a previous study, we demonstrated a dietary intervention with FF*/Bb*-containing synbiotic blend and a mixture of four short specific BLG-derived peptides to partially induce OT for the whole whey protein (35). However, the underlying process associated with the preventive capacity of these BLG-peptides and dietary adjunct treatment is not yet understood. Previous studies investigating the antigen-specific approach with protein fragments and/or the immunomodulatory effects of dietary components (e.g., poly-unsaturated fatty acids, non-digestible oligosaccharides, probiotic bacteria, synbiotics) have suggested the involvement of CD25^+^Foxp3^+^ T_regs_ in the suppression of the allergic response to cow’s milk (30, 42–44). Therefore, in the current study, the same CMA mouse model was used to confirm the capacity of BLG-peptides and FF/*Bb* synbiotics to partially prevent the allergic skin response to whole whey. Furthermore, the effects on gut immune parameters and T cell phenotype directly after the OT induction phase were assessed.

## Materials and Methods

### Animals

Three-week-old specific pathogen-free female C3H/HeOuJ mice were purchased from Charles River Laboratories (Sulzfeld, Germany) and housed in the animal facility of Utrecht University. The animal use was approved by the Animal Ethics Committee of Utrecht University and the Central Commission for Animal use (approval numbers DEC 2013.II.11.116 and AVD108002015262). Animal care and use were conducted in accordance with the guidelines of the Animal Ethics committee of Utrecht University.

### Peptides and Diet

Four synthetic peptides, consisting of 18-AA from the B variant of BLG, were purchased from JPT Peptide Technologies (Berlin, Germany). Each of the four peptides had a 12-AA overlap with the preceding sequence (35, 36). A mixture of the four BLG-derived peptides was prepared by dissolving the lyophilized peptides in sterile phosphate-buffered saline (PBS; Lonza, Walkersville, MD, USA) until each peptide in the mixture was at a concentration of 0.08 mg/mL (further referred to as PepMix).

CMP-free, AIN-93G-based standard mouse chow was composed (control diet) by Research diet Services (RDS, Wijk bij Duurstede, The Netherlands) and supplemented with 1% (wt:wt) of non-digestible scFOS (Raftilose P95, Beneo Orafti S.A., Oreye, Belgium) and lcFOS (Raftiline HP, Beneo Orafti S.A.) in a ratio 9:1 and 2% (wt:wt) 2 × 10^9^ CFU/g *B. breve* M-16V (Morinaga Milk Industry, Tokyo, Japan) (FF/*Bb* diet). The synbiotic components were mixed through the diet where FF was exchanged for cellulose and *Bb* for maltodextrin and pressed into pellets. Diets were stored at 4°C prior to use.

### OT Induction in a Mouse Model of Orally Induced CMA

**Figure 1:**
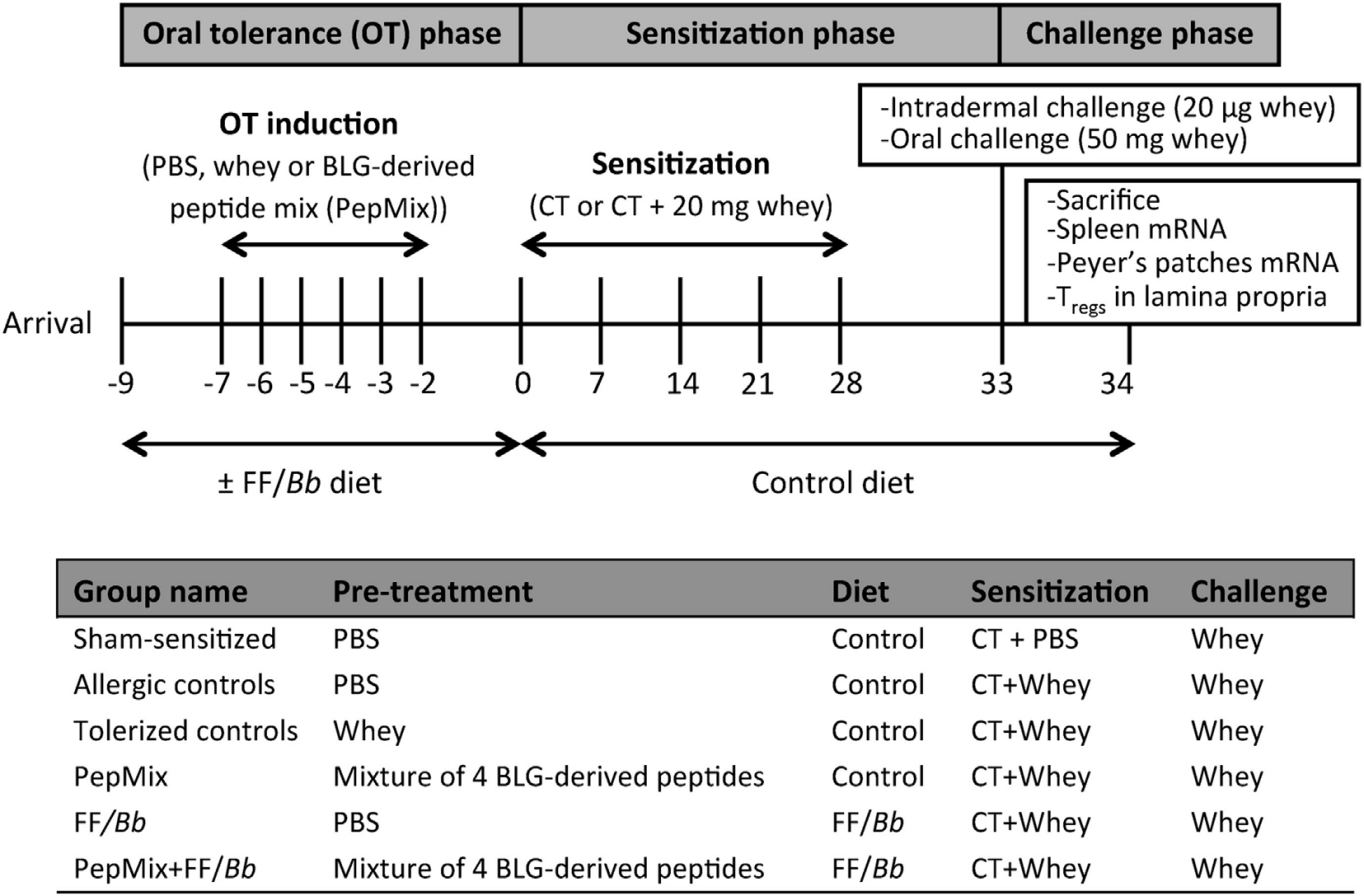
A schematic overview of the murine model for CMA prevention with the oral tolerance (OT), sensitization, and challenge phases.

**Figure 2:**
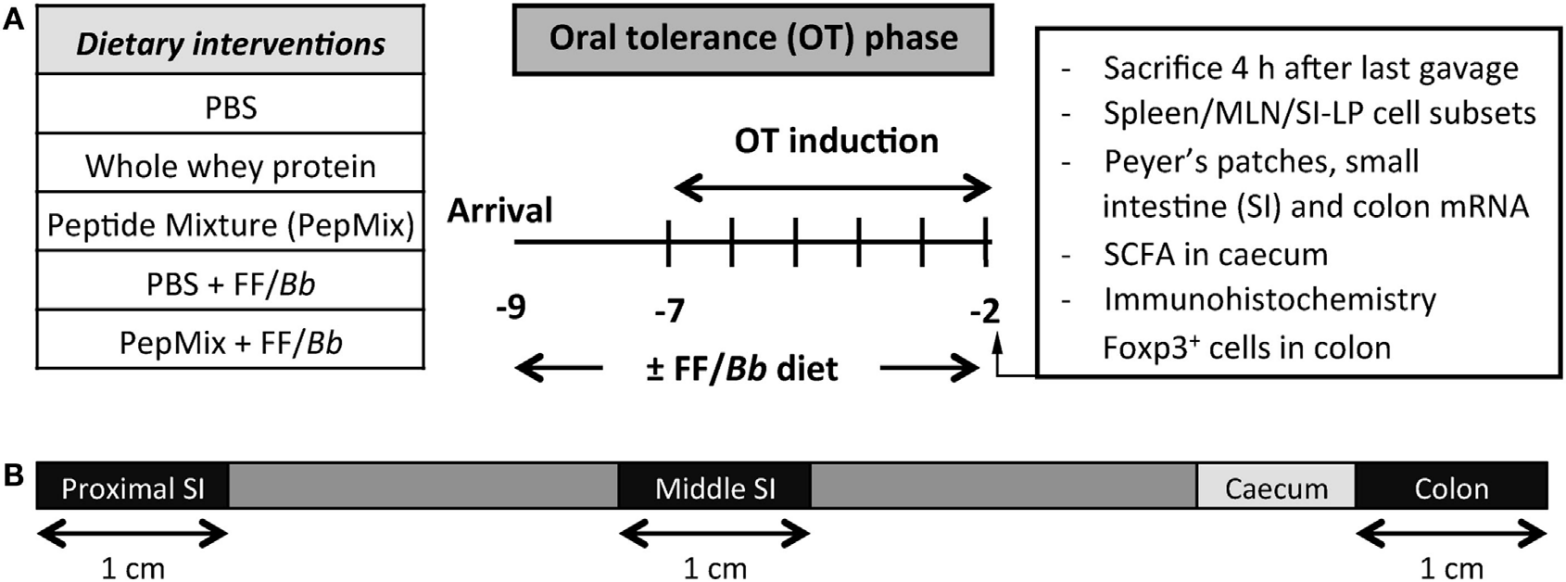
**(A)** A schematic overview of the study investigating the impact of the dietary interventions during the OT phase. **(B)** A schematic overview of the intestine indicating the different parts used for qPCR analysis.

Upon arrival, mice were fed the control or the FF/*Bb* diet *ad libitum* for a period of 9 days. During the OT phase, they received daily oral gavages with 0.5 mL PBS, PepMix (total 0.16 mg peptides/0.5 mL PBS; 0.04 mg of each BLG-derived peptide) or whole whey protein as a positive control for maximum OT induction (50 mg/0.5 mL PBS; DMV International, Veghel, The Netherlands) for a period of 6 days. In the first study (Figure 1), following the OT phase, all mice were fed the control diet and orally sensitized for five consecutive weeks with 10 µg cholera toxin (List Biological Laboratories, Inc., CA, USA) as an adjuvant with or without 0.5 mL homogenized whey (40 mg whey/mL PBS). Five days after the last sensitization, mice were challenged intradermally (i.d.) with whey protein and the acute allergic skin response and signs of anaphylaxis were assessed. The same day, mice were orally challenged with 50 mg whey/0.5 mL PBS and were sacrificed by cervical dislocation 18 h thereafter. In the second study (Figure 2), mice were sacrificed 4 h after the last OT administration to assess markers of immunomodulation during the OT phase.

### Assessment of the Allergic Response

To measure the potential allergic response to whey, mice were i.d. challenged in the ear pinnae of both ears with 10 µg whey in 20 µL PBS and the acute allergic skin response [expressed as the Δ ear swelling (μm)] was evaluated. The ear thickness prior to and 1 h after the i.d. challenge of isoflurane-anesthetized mice was measured in duplicate for each ear using a digital micrometer (Mitutoyo, Veenendaal, The Netherlands). To express the net ear swelling, the average basal ear thickness was subtracted from the average ear thickness at 1 h after i.d. challenge. In addition to the acute allergic skin response, the anaphylaxis was scored according to Schouten et al. (45).

### Cell Isolation from Tissues

Lymphocytes were isolated from the spleen, MLN, and small intestine lamina propria (SI-LP) of mice that were sacrificed just after the OT period (Figure 2). SI-LP single-cell suspensions were also prepared from mice that were sensitized and challenged after the OT period (Figure 1). MLN cell suspensions were prepared as previously described (46). Single-cell splenocyte suspensions were obtained by passing the organs through 70 µm cell strainers and lysing the red blood cells with a buffer containing 8.3 g/L NH_4_Cl, 1 g/L KHCO_3_, and 37.2 mg/L EDTA. Cells from the SI-LP were isolated from the whole small intestine (SI) after removing the Peyer’s patches (PP), washing the intestine in cold PBS, opening it longitudinally, and mincing it in 0.5 cm fragments. To remove epithelial cells and intraepithelial lymphocytes, intestinal fragments were washed in Hank’s Balanced Salt Solution (HBSS; Invitrogen) containing 15 mM HEPES (Gibco, Life Technologies, Carlsbad, CA, USA), pH = 7.2 and incubated 4 × 15 min with HBSS supplemented with 15 mM HEPES, 5 mM Na_2_-EDTA, 10% FCS, and penicillin (100 U/mL)/streptomycin (100 µg/mL), pH = 7.2. Then, samples were washed in RPMI 1640, 5% FCS and penicillin (100 U/mL)/streptomycin (100 µg/mL) and digested for 2 × 45 min with an enzyme solution containing 0.5 mg/mL Collgenase type VIII (Sigma-Aldrich). Fragments were vortexed for 10 s after each incubation and poured over a 70 µm cell strainer while collecting the passing cells. SI-LP lymphocytes were purified using a Percol gradient (pH = 7.2). Spleen, MLN, and SI-LP cell suspensions were resuspended in PBS/1% BSA and used for flow cytometric analysis.

### DC and T Cell Subsets Analyzed by Flow Cytometry

Single-cell suspensions from SI-LP, MLN, and/or spleen were obtained and incubated for 15 min with anti-mouse CD16/CD32 (Mouse BD Fc Block; BD Pharmingen, San Jose, CA, USA) in PBS + 1% BSA + 5% FBS buffer to block non-specific binding sites. The following monoclonal antibodies were used according to the manufacturer’s instructions to stain surface markers on DC in the MLN and SI-LP of mice sacrificed in the OT phase: CD11c-PerCp-Cy5.5, CD11b-PE, MHC class II-PE-Cy7, CD103-APC or PE, CD8α-FITC, CD40-FITC, CD80-APC, CD86-FITC, and F4/80-APC-Cy7 (all from eBiosciences, San Diego, CA, USA). For analyzing T cell subsets in all three tissues of the OT mice and the SI-LP of whey-sensitized and challenged mice, cells were first extracellularly stained with CD4-PerCp-Cy5.5, and CD25-AlexaFluor488 (all from eBiosciences). For intracellular staining, cells were first fixated and permeabilized with Foxp3 Staining Buffer Set (eBioscience) according to manufacturer’s protocol followed by incubation with Tbet-PE, GATA3-PE or Foxp3-PE-Cy7 antibody (eBioscience). Dead cells were excluded by the use of fixable viability dye eFluor780 (eBioscience), except in the SI-LP analysis of whey-sensitized mice where dead cells were excluded by forward/side scatter gating. Results were collected with BD FACSCanto II flow cytometer (Becton Dickinson, Franklin Lakes, NJ, USA) and analyzed with FlowLogic software (Inivai Technologies, Mentone, VIC, Australia).

### qPCR Analysis of Gene Expression

PP and spleen samples from whey-sensitized mice (Figure 1) and PP and intestinal samples from the mice sacrificed during the OT phase (Figures 2) were collected in RNA*later* (Invitrogen, Thermo Fisher Scientific, Waltham, MA, USA) and stored at −20°C until cDNA synthesis. mRNA was extracted using a RNeasy Mini Kit (Qiagen, Hilden, Germany) and cDNA was synthesized using an iScript™ cDNA synthesis kit (Bio-Rad, Basel, Switzerland) according to the manufacturer’s protocol. Quantitative analysis was performed on a CFX96 real-time PCR detection system with the use of an iQ SYBR™ Green PCR supermix (both from Bio-Rad). Commercially available primers for Foxp3, Tbet, GATA3, RorγT, IL-10, galectin-9, TGF-β, IL-13, IFN-γ, and IL-22 were obtained (all from Qiagen). Beta-actin (B-actin) (Qiagen) was used as a reference gene for the samples from the whey-sensitized mice and RPS13 (Qiagen) was used for the samples obtained from mice during the OT phase. Relative mRNA expression was calculated as 100 × 2^Ct[reference gene] − Ct[gene of interest]^ (47).

### SCFA Concentrations in Cecum

The cecal content was collected from mice sacrificed during the OT phase, immediately frozen in liquid nitrogen and stored at −80°C until measurement. Samples were defrosted on ice, homogenized and centrifuged for 10 min at 14,000 rpm. Concentration of acetic, propionic, butyric, and valeric acid were determined in the cecal supernatants by means of a Shimadzu GC2010 gas chromatograph as previously described (48), using 2-ethylbutyric acid as an internal standard.

### Immunohistochemistry Foxp3^+^ Cells in Colon

The colon from mice sacrificed during the OT phase was collected and rolled in a swiss roll as previously described for the SI (46, 49). The swiss rolls were fixed in 10% v/v formalin for at least 48 h before embedding them in paraffin (Leica IG1150C; Leica Microsystems, Rijswijk, The Netherlands). 5 µm Thick sections were sliced on a microtome (Leica Microsystems) and mounted on coated slides. Sections were first dewaxed, microwaved for 13 min in 0.01 M sodium citrate buffer and left to cool down for 30 min. Non-specific binding was blocked by 30 min incubation with 5% rabbit serum (DAKO, Heverlee, Belgium) in 1% BSA/PBS. Samples were incubated overnight at 4°C with 10 µg/mL rat anti-mouse Foxp3 purified antibody or rat IgG2a isotype as control in 1% BSA/PBS (both from eBioscience). After washing, slides were incubated with 3% H_2_O_2_ for 30 min in the dark followed by 1 h-incubation at room temperature with rabbit anti-rat-biotinylated antibody (Jackson ImmunoResearch, West Grove, PA, USA) in 1% BSA/PBS. Finally, avidin-biotin complex (Vector Laboratories, Peterborough, UK) was added for 1 h before developing the color with 3,3′-diaminobenzidine (Sigma-Aldrich) and counterstaining with hematoxylin. Slides were dehydrated and covered using Pertex mounting medium (Histolab, Goteborg, Sweden) and a cover glass. To enumerate the positive cells, Leica microscope was used and results are presented as number of cells per crypt unit multiplied by 100.

### Statistical Analysis

All data were analyzed using GraphPad Prism 6.0 software for Macintosh (GraphPad Software, San Diego, CA, USA). For the analysis of the anaphylactic shock scores, the non-parametric Kruskal–Wallis test followed by a Dunn’s *post hoc* test for selected comparisons was used. All other data sets were analyzed by one-way ANOVA, followed by a Bonferroni’s multiple comparison *post hoc* test for selected comparisons. To meet ANOVA’s criteria for normal data distribution and equality of variance between experimental groups, transformation was applied if required. If this failed, the non-parametric Kruskal–Wallis test followed by a Dunn’s *post hoc* test for selected comparisons was applied. The non-parametric Spearman correlation coefficient test was used to test for correlation. Data are presented as mean ± SEM or Tukey box-and-whisker plots of five to eight animals per group for the mice sensitized and challenged with whey, and four to six per group for the mice sacrificed during the OT phase. *p* < 0.05 was considered of statistical significance.

## Results

### Reduced Allergic Skin Response to Whole Whey Protein in Mice Pretreated with BLG-Derived Peptides while Fed FF/*Bb* Synbotics Prior to Sensitization with Whey

**Figure 3:**
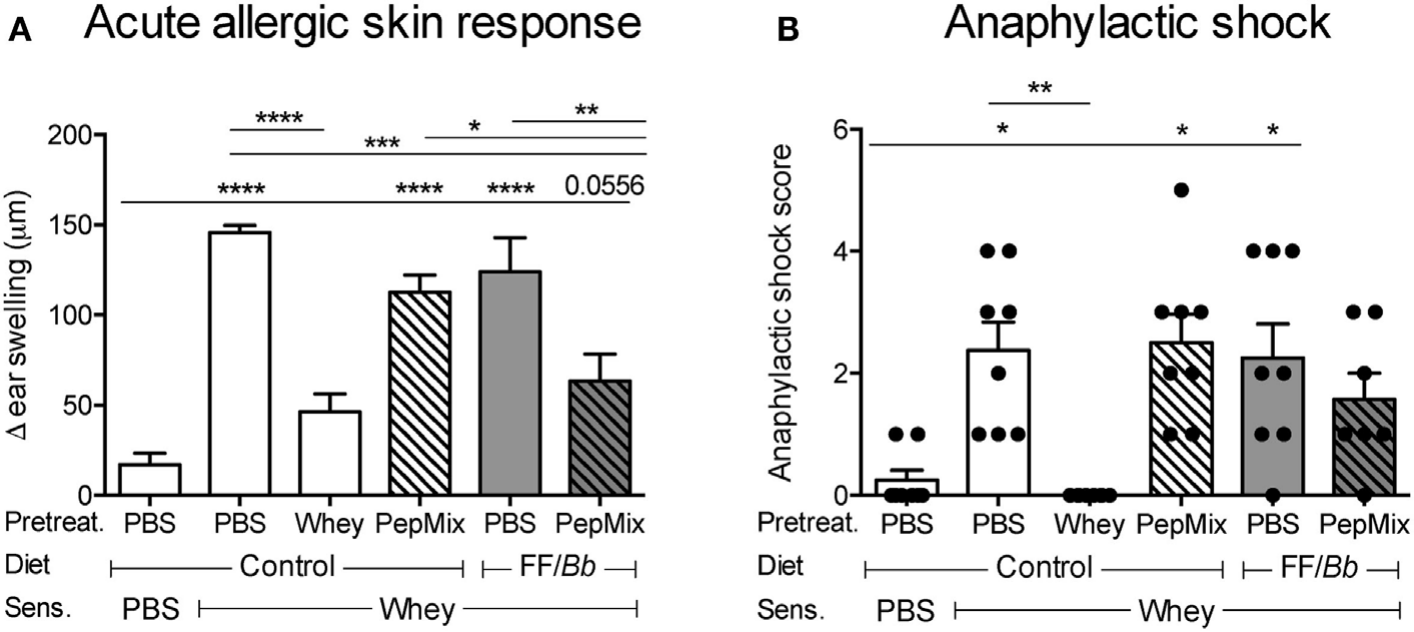
Effect of dietary interventions with β-lactoglobulin (BLG)-derived peptides and/or FF/*Bb* synbiotics on the prevention of CMA. The acute allergic skin response **(A)** and signs of anaphylaxis **(B)** were recorded upon i.d. whey challenge in the ear pinnae. Data are presented as mean ± SEM; *n* = 6–8/group; *p < 0.05, **p < 0.01, ***p < 0.001, ****p < 0.0001; **(A)** was analyzed with one-way ANOVA followed by Bonferroni’s *post hoc* test for selected groups; **(B)** was analyzed with Kruskal–Wallis’ non-parametric test, followed by Dunn’s *post hoc* test for selected pairs.

As expected, mice that were fed whole whey protein before sensitization with whey developed tolerance to whey and were prevented from allergic symptoms upon i.d. whey challenge, therefore referred to as the tolerant controls (Figures 1,3A). Similarly, exposing mice to the combination of PepMix+FF*/Bb* before sensitization prevented them from developing acute allergic skin response to whole whey (Figure 3A). The combination of PepMix and FF*/Bb* significantly prevented the ear swelling response while PepMix or FF*/Bb* alone did not, indicating that the FF/*Bb* diet facilitates the tolerogenic properties of the PepMix. Furthermore, whey-pretreated and PepMix+FF*/Bb*-pretreated mice did not significantly differ from the sham-sensitized mice in anaphylactic symptoms score (Figure 3B) and ear swelling (Figure 3A) while PepMix or FF/*Bb* alone enhanced the allergic response compared to the sham-sensitized mice.

### The Reduced Allergic Response Was Associated with Increased T Cell Activation and Induction of T_regs_ in the SI-LP

**Figure 4:**
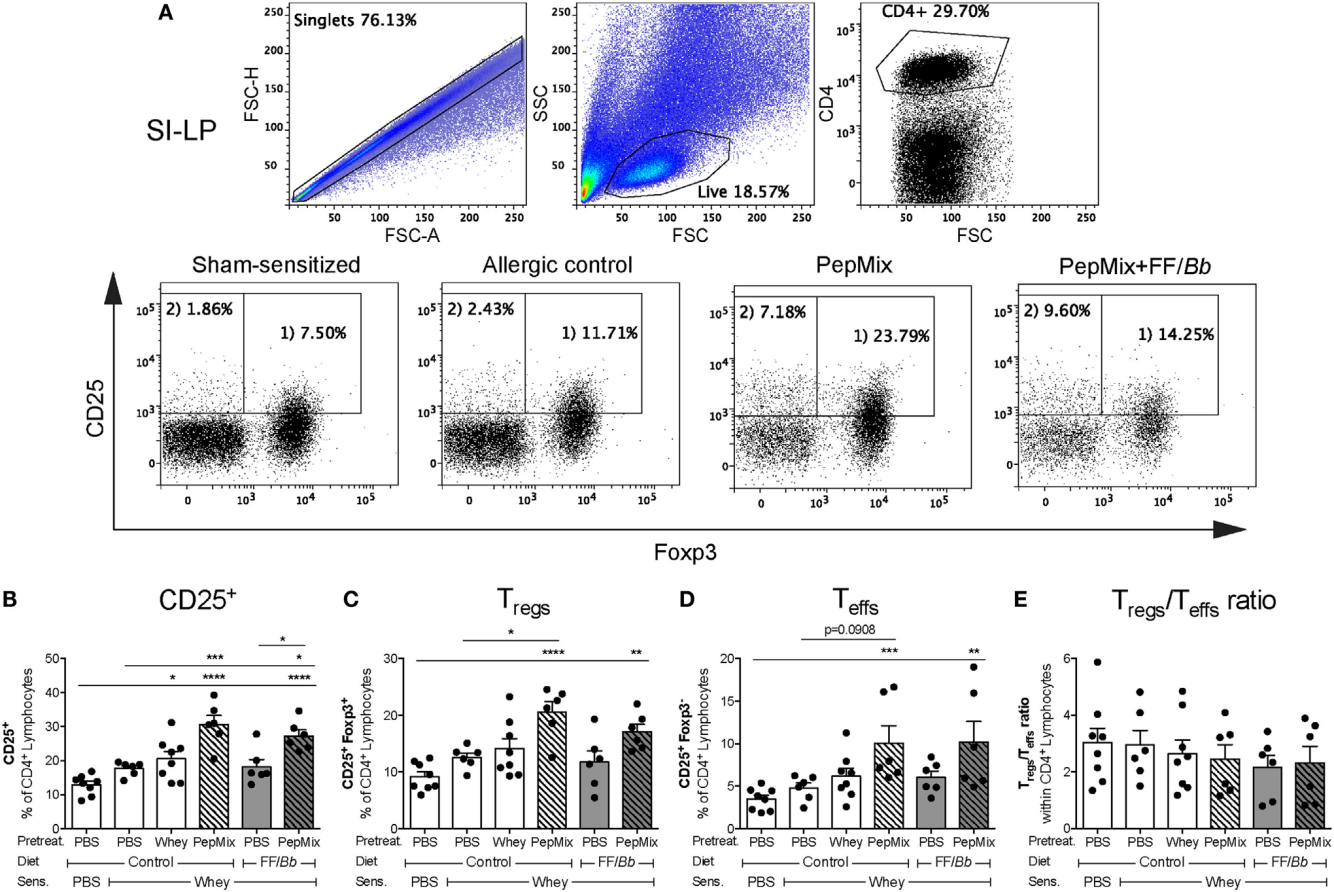
Effect on T_regs_ percentage in the small intestine lamina propria (SI-LP) in the challenge phase of the CMA model. Mice were sacrificed 18 h after oral challenge and SI-LP cells were analyzed by flow cytometry. Representative FACS plots are presented in **(A)**. Percentages of activated CD25^+^ T cells **(B)**, T_regs_ **(C)**, and T_effs_ **(D)** were determined in the CD4^+^ lymphocyte population. T_regs_/T_effs_ ratio in the SI-LP was also calculated **(E)**. Data are presented as mean ± SEM of *n* = 6–8/group; *p < 0.05, **p < 0.01, ***p < 0.001, ****p < 0.0001 one-way ANOVA followed by Bonferroni’s *post hoc* test for selected groups.

In order to investigate whether the prevention of CMA symptoms in the whey allergic mice is associated with changes in T_regs_ numbers, SI-LP cells were isolated after the challenge phase and analyzed by flow cytometry (Figure 4A). Interestingly, mice that received either the whole whey protein, the PepMix, or the PepMix+FF/*Bb* before the sensitization showed increased numbers of CD25^+^ (activated) T cells in the SI-LP compared to the sham-sensitized animals (Figure 4B). The CD25^+^ T cells in the PepMix and PepMix+FF/*Bb* groups were also significantly more than in the allergic control mice. Similarly, PepMix and PepMix+FF/*Bb* enhanced CD25^+^Foxp3^+^ T_regs_ (Figure 4C) as well as CD25^+^Foxp3^−^ effector T cells (T_effs_) (Figure 4D) compared to the sham-sensitized mice, while only the number in the PepMix group was different compared to the allergic control. As both T_regs_ and T_effs_ populations were increased, the balance between those cell types remained unaffected by the dietary intervention (Figure 4E).

### Exposure to PepMix+FF/*Bb* before Sensitization Leads to T_h_1 Markers Increase in the PP after the Challenge Phase

**Figure 5:**
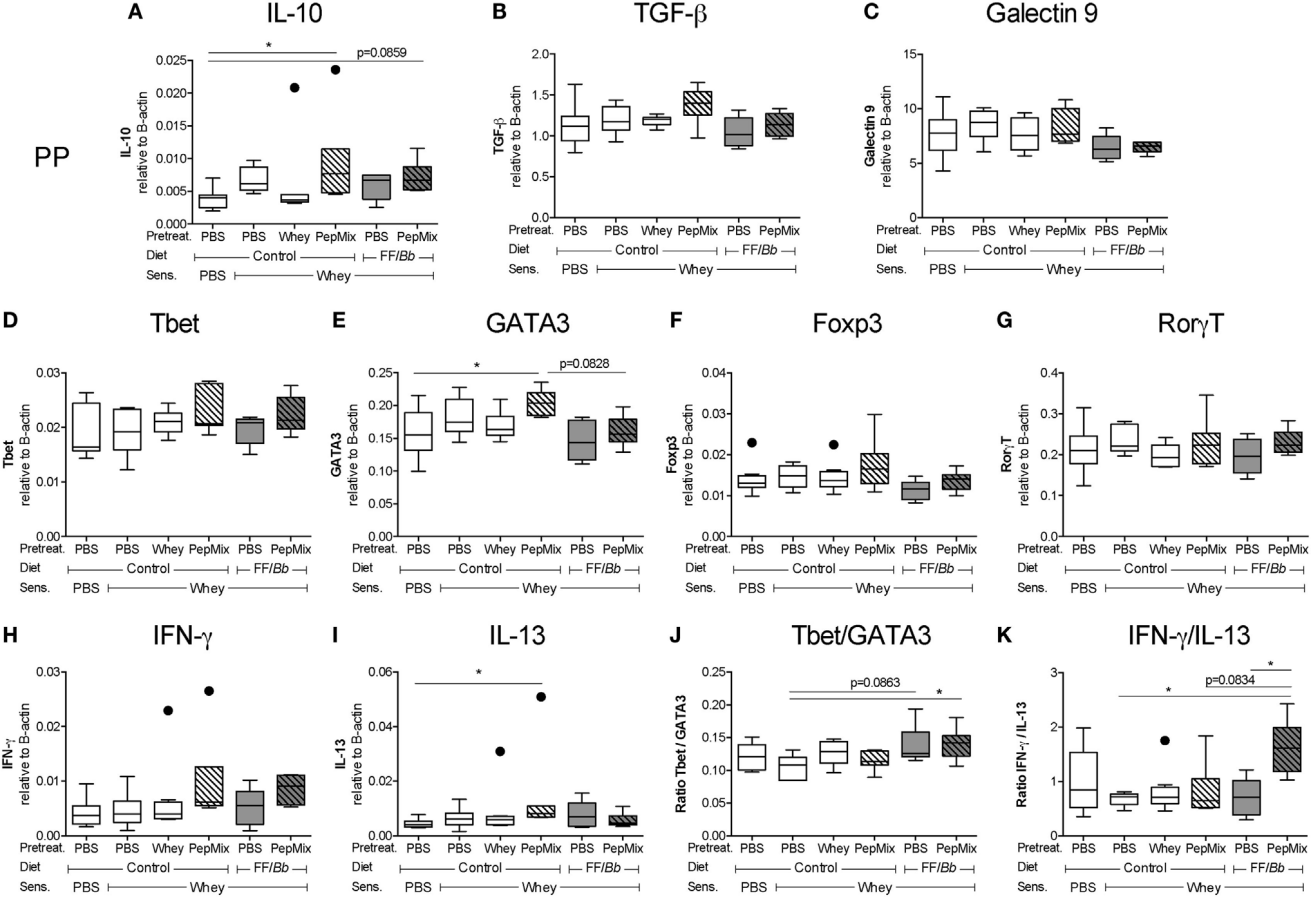
mRNA expression in the Peyer’s patches (PP) of whey-sensitized mice in the challenge phase. PP of mice were excised 18 h after the oral challenge with whey, and used for mRNA isolation and cDNA synthesis. mRNA expression of the regulatory IL-10 **(A)**, TGF-β **(B)**, and galectin-9 **(C)** markers, Tbet **(D)**, GATA3 **(E)**, Foxp3 **(F)**, and RorγT **(G)** transcription factors and T_h_1-associated IFN-γ **(H)** and T_h_2-associated IL-13 **(I)** were measured using a real-time qPCR. The Tbet/GATA3 **(J)** and IFN-γ/IL-13 ratios **(K)** were calculated to represent the T_h_1/T_h_2 balance in the PP. mRNA expression levels were normalized to the B-actin housekeeping gene expression. Data are presented as box-and-whisker Tukey plots, *n* = 6–8/group; * *p* < 0.05 one-way ANOVA followed by Bonferroni’s *post hoc* test for selected groups. Panels **(A,D,I)** were analyzed by Kruskal–Wallis’ non-parametric test, followed by Dunn’s *post hoc* test for selected pairs.

The impact of the interventions on the gene expression of regulatory molecules in the PP was studied. IL-10 mRNA expression in the PepMix-fed mice was increased compared to the sham-sensitized group, and a similar tendency was observed in the PepMix+FF*/Bb* group (Figure 5A), while TGF-β and galectin-9 remained unchanged (Figures 5B,C). On the other hand, PepMix also increased GATA3 mRNA expression compared to sham-sensitized mice while combining PepMix with FF/*Bb* diet tended to prevent this increase (*p* < 0.10) (Figure 5E). A similar pattern was observed for the mRNA expression of IL-13 (Figure 5I). Expression of the Tbet, Foxp3, and RorγT transcription factors and IFN-γ cytokine remained unaffected (Figures 5D,F,G,H). However, in PepMix+FF/*Bb*-fed mice, the ratio of Tbet/GATA3 and IFN-γ/IL-13 was increased compared to the allergic control mice (Figures 5J,K) and the IFN-γ/IL-13 ratio was higher also when compared to FF/*Bb* alone (Figure 5K). These effects indicate a shift to a more T_h_1 predominance in these mice (Figure 5D). Only limited changes were observed in the spleen (Figure S1 in Supplementary Material).

### FF/*Bb* Enhances Cecal Propionic Acid Concentrations during the OT Phase Which Correlates with Foxp3^+^ Cells in the Colon

**Figure 6:**
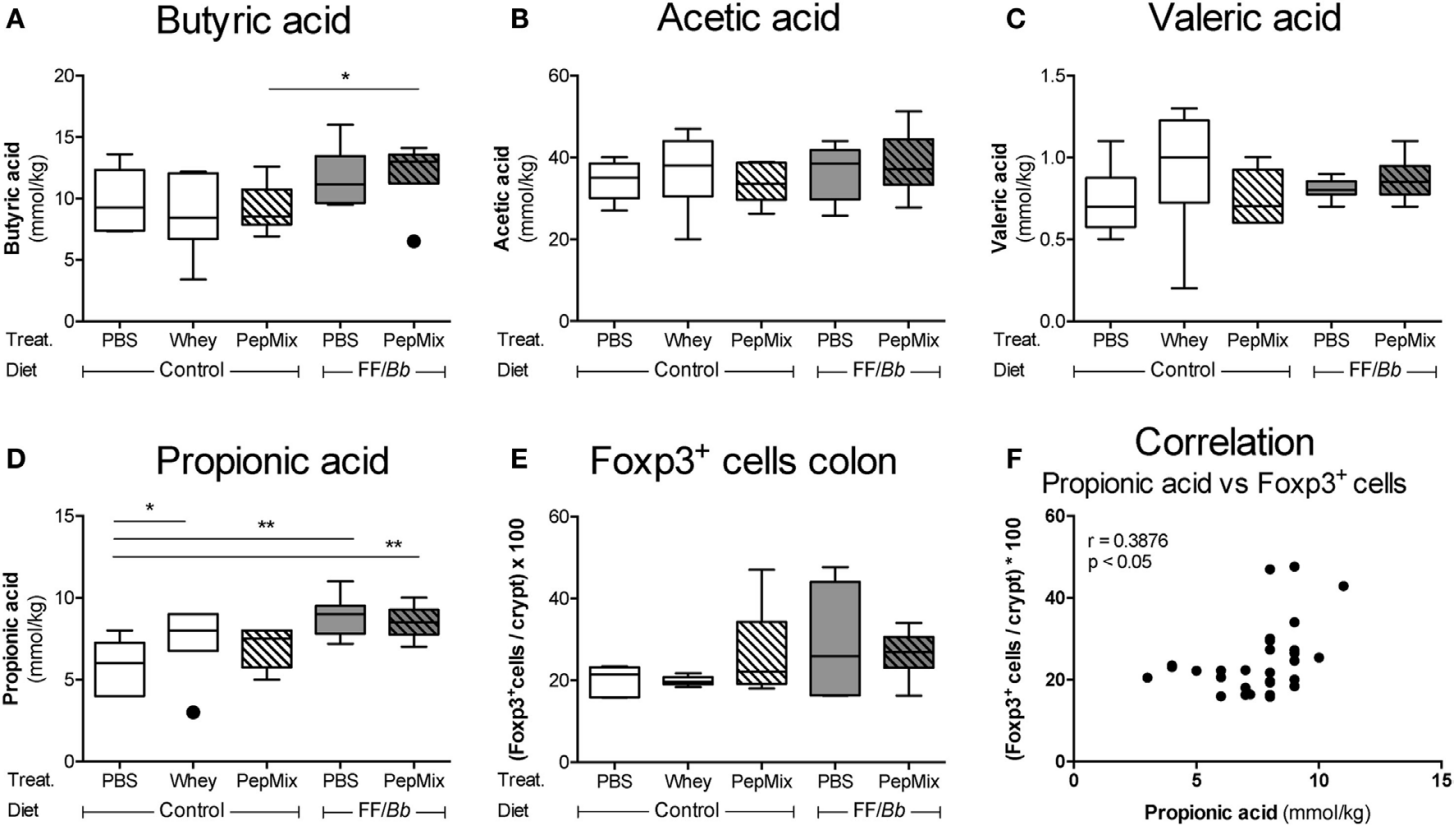
Early effects of orally administered β-lactoglobulin-peptides and dietary FF/*Bb* synbiotics on short-chain fatty acid (SCFA) production in the cecum and Foxp3^+^ cells in the colon during the oral tolerance (OT) phase. Effect of the diet on the SCFA concentrations in the cecum **(A–D)** and on the frequency of immunohistochemically stained Foxp3^+^ cells in the colon **(E)**. Propionic levels in the cecum were found to positively correlate with the frequency of Foxp3^+^ cells in the colon **(F)**. Data are presented as box-and-whisker Tukey plots, *n* = 6/group; *p < 0.05, **p < 0.01 one-way ANOVA followed by Bonferroni’s *post hoc* test for selected groups after excluding the outliers as indicated by Tukey. Panel **(F)** was analyzed with the non-parametric Spearman correlation coefficient test.

To examine the effects of the dietary interventions on the early markers of tolerance, samples were collected directly after the 6-day OT exposure to whole whey, PBS, PepMix, FF/*Bb*, or PepMix+FF/*Bb* (sacrifice 4 h after the last oral administration) (Figure 2A). As dietary interventions may influence the microbiota colonization in the gut as well as its metabolism (50), the concentrations of microbiota metabolites SCFA were measured in the cecal content. Propionic acid increased in mice fed whole whey protein or FF/*Bb* either or not combined with PepMix (Figure 6D). Furthermore, FF/*Bb* increased butyrate concentrations in PepMix-exposed mice compared to PepMix mice fed the control diet (Figure 6A). Other SCFA, such as acetic and valeric acids, were not affected by the dietary intervention (Figures 6B,C). As changes in the microbiota or SCFA production are suggested to influence the development of T_regs_ (51, 52), immunohistochemical staining of Foxp3 cells was performed in the colonic tissue. Although the numbers of Foxp3^+^ cells in the colon were not significantly affected (Figure 6E), they showed a similar pattern and correlated positively with the propionic levels in the cecum (Figure 6F).

### PepMix+FF/*Bb* Enhances T_regs_ over T_effs_ Markers in the PP during OT Phase

**Figure 7:**
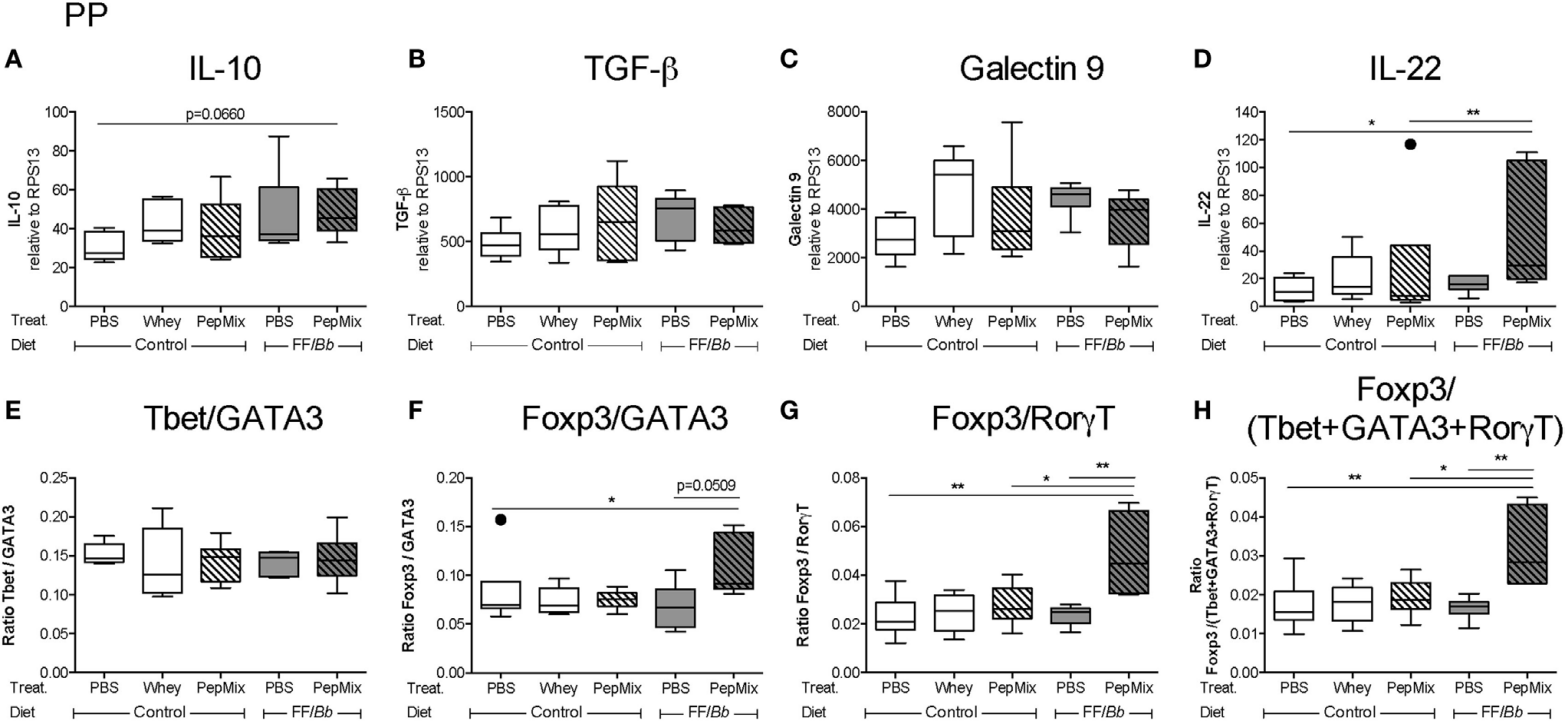
Impact of the dietary interventions on mRNA expression in the Peyer’s patches (PP) during the oral tolerance (OT) phase. PP were collected 4 h after the last oral administration of the OT phase in order to study the mRNA expression of potential regulatory markers, such as IL-10 **(A)**, TGF-β **(B)**, galectin-9 **(C)**, and IL-22 **(D)**. Also changes in the T_h_ cell marker balance were studied **(E–H)**. mRNA expression was normalized to the RPS13 housekeeping gene expression. Data are presented as box-and-whisker Tukey plots, *n* = 5–6/group; *p < 0.05, **p < 0.01 one-way ANOVA followed by Bonferroni’s *post hoc* test for selected groups after excluding outliers as indicated by Tukey. Panels **(A,F)** were analyzed by Kruskal–Wallis’ non-parametric test, followed by Dunn’s *post hoc* test for selected pairs.

In the PP, PepMix+FF/*Bb* resulted in increased mRNA expression of the T_h_17-suppressive and epithelial cell-protective cytokine IL-22 (53, 54) compared to both the PBS control mice as well as to mice fed PepMix alone (Figure 7D). IL-10 in this group followed the same tendency when compared to the PBS control (*p* < 0.10) (Figure 7A). Also Foxp3 (T_regs_) mRNA expression tended to increase in the PepMix+FF/*Bb* mice (*p* < 0.10), while the expression of T_effs_-associated transcription factors, such as Tbet, GATA3, and RorγT, remained unaffected (Figure S2A in Supplementary Material). These effects resulted in an increased ratio of the T_regs_ marker Foxp3 over the T_h_2 marker GATA3 (Figure 7F) or the T_h_17 marker RorγT (Figure 7G) in the PepMix+FF/*Bb*-fed mice compared to the PBS control. No effect of the dietary interventions was distinguished on TGF-β and galectin-9 expression (Figures 7B,C). Similarly, the Tbet/GATA3 ratio was unaffected at this early stage (Figure 7E). However, the ratio of Foxp3 over the T_effs_-associated Tbet, GATA3, and RorγT was significantly increased in the PepMix+FF/*Bb* group compared to the PBS control as well as to the PepMix or FF/*Bb* alone (Figure 7H).

### PepMix+FF/*Bb* Enhances Foxp3^+^ T_h_ Cells in the MLN during the OT Phase, while Reducing the Frequency of GATA3^+^ and T_effs_ Cells

**Figure 8:**
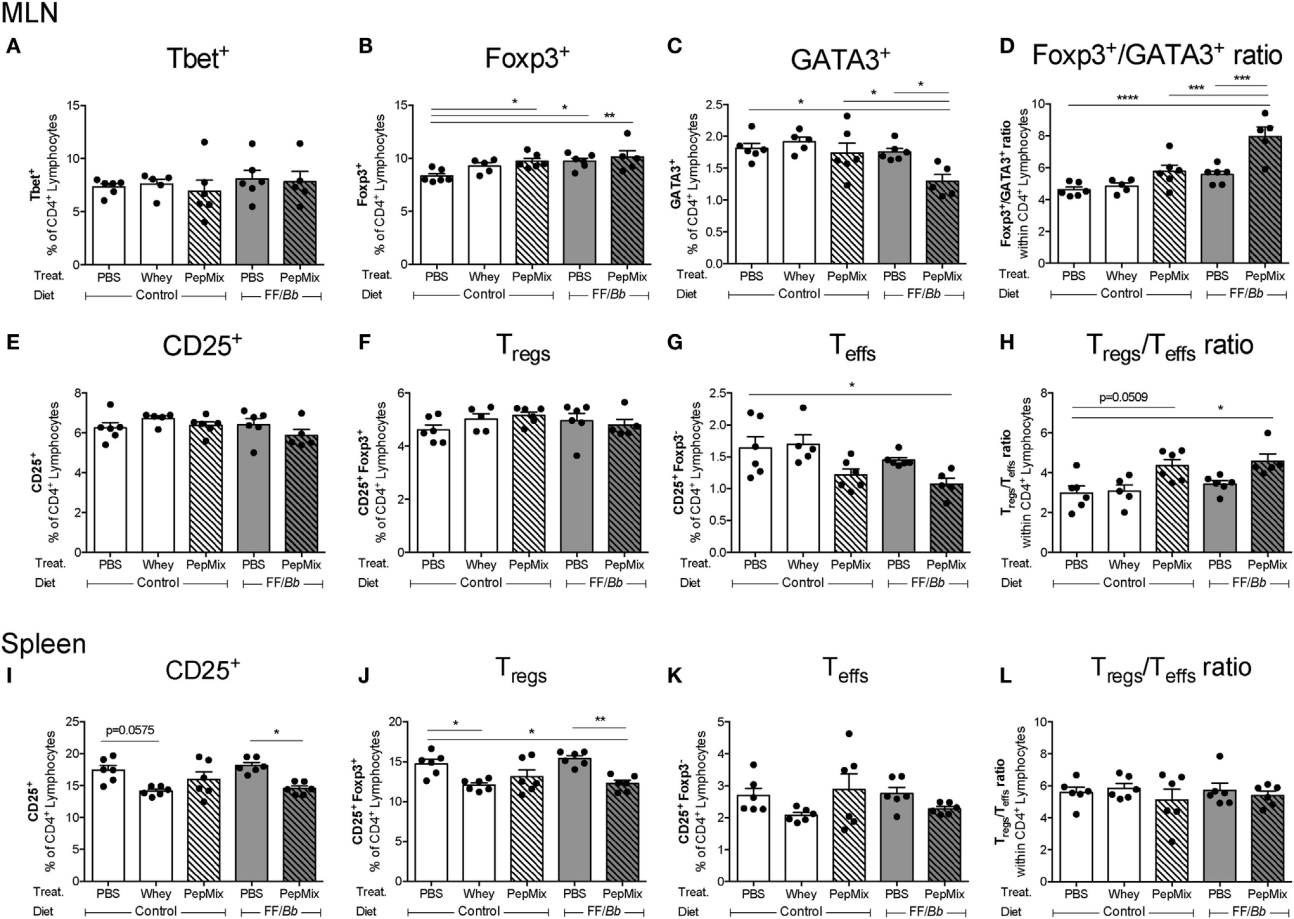
Effect of the interventions on the percentage of T cell subsets in the mesenteric lymph nodes (MLN) and spleen during the oral tolerance (OT) phase. T cell subsets were studied in the draining MLN **(A–H)** and spleen **(I–L)** of mice sacrificed after the OT phase. Tbet^+^ **(A)**, Foxp3^+^ **(B)**, and GATA3^+^ **(C)** are presented as percentage of the CD4^+^ population. The PepMix+FF/*Bb* intervention increased the ratio of Foxp3^+^ over GATA3^+^ cells **(D)**. CD4^+^ T cells were also analyzed for their expression of CD25 **(E,I)** and Foxp3 and the resulting percentages of T_regs_ **(F,J)** and T_effs_ **(G,K)** are presented. PepMix+FF/*Bb* enhanced the T_regs_/T_effs_ ratio within the MLN **(H)**, but not in the spleen **(L)**. Data are presented as mean ± SEM of *n* = 5–6/group for the MLN and *n* = 6/group for the spleen; *p < 0.05, **p < 0.01, ***p < 0.001, ****p < 0.0001 one-way ANOVA followed by Bonferroni’s *post hoc* test for selected groups. Panels **(A,B,E,H,I)** were analyzed by Kruskal–Wallis’ non-parametric test, followed by Dunn’s *post hoc* test for selected pairs.

In the MLN, a major inductive site in the gut, interventions with PepMix or FF/*Bb* alone or in combination (PepMix+FF/*Bb*) enhanced the frequency of Foxp3^+^ T_h_ cells during the OT phase (Figure 8B). However, the frequency of GATA3^+^ T_h_2 cells was reduced only in the combined PepMix+FF/*Bb* group (Figure 8C), resulting in a Foxp3^+^ predominating over GATA3^+^ T_h_ cells when compared to the PBS control as well as the groups fed PepMix or FF/*Bb* alone (Figure 8D). The percentage of Tbet^+^ Th1 cells remained unaffected (Figure 8A). Interestingly, the percentage of activated CD25^+^ T cells was low in the PepMix+FF/*Bb* group (Figure 8E) and this reached significance when gated for CD25^+^Foxp3^−^ T_effs_ (Figure 8G). This enhanced the ratio of T_regs_ over T_effs_ in PepMix-exposed mice fed the FF/*Bb* diet (Figure 8H). Even though the percentages of CD25^+^Foxp3^+^ T_regs_ were not affected by the interventions (Figure 8F), more CD4^+^CD25^+^ cells expressed the Foxp3 transcription factor (data not shown). Exposing mice to PepMix alone also increased the percentage of Foxp3^+^ cells but only tended to increase the T_regs_/T_effs_ cell ratio (*p* < 0.10) (Figure 8H). In the spleen, on the other hand, T_regs_ were reduced in both whey-tolerized as well as in PepMix+FF/*Bb-*fed mice compared to the PBS control (Figure 8J). Similarly, activated CD25^+^ lymphocytes were decreased when FF*/Bb*-fed mice were also administered the PepMix during the OT period (Figure 8I). However, no effect of the interventions was observed on the percentage T_effs_ (Figure 8K) or the T_regs/_T_effs_ ratio (Figure 8L). In addition, no effects on T_regs_ and T_effs_ were observed in the SI-LP (Figure S3 in Supplementary Material).

### Feeding PepMix+FF/*Bb* Enhances CD11b^+^ over CD11b^−^ DC in the SI-LP

**Figure 9:**
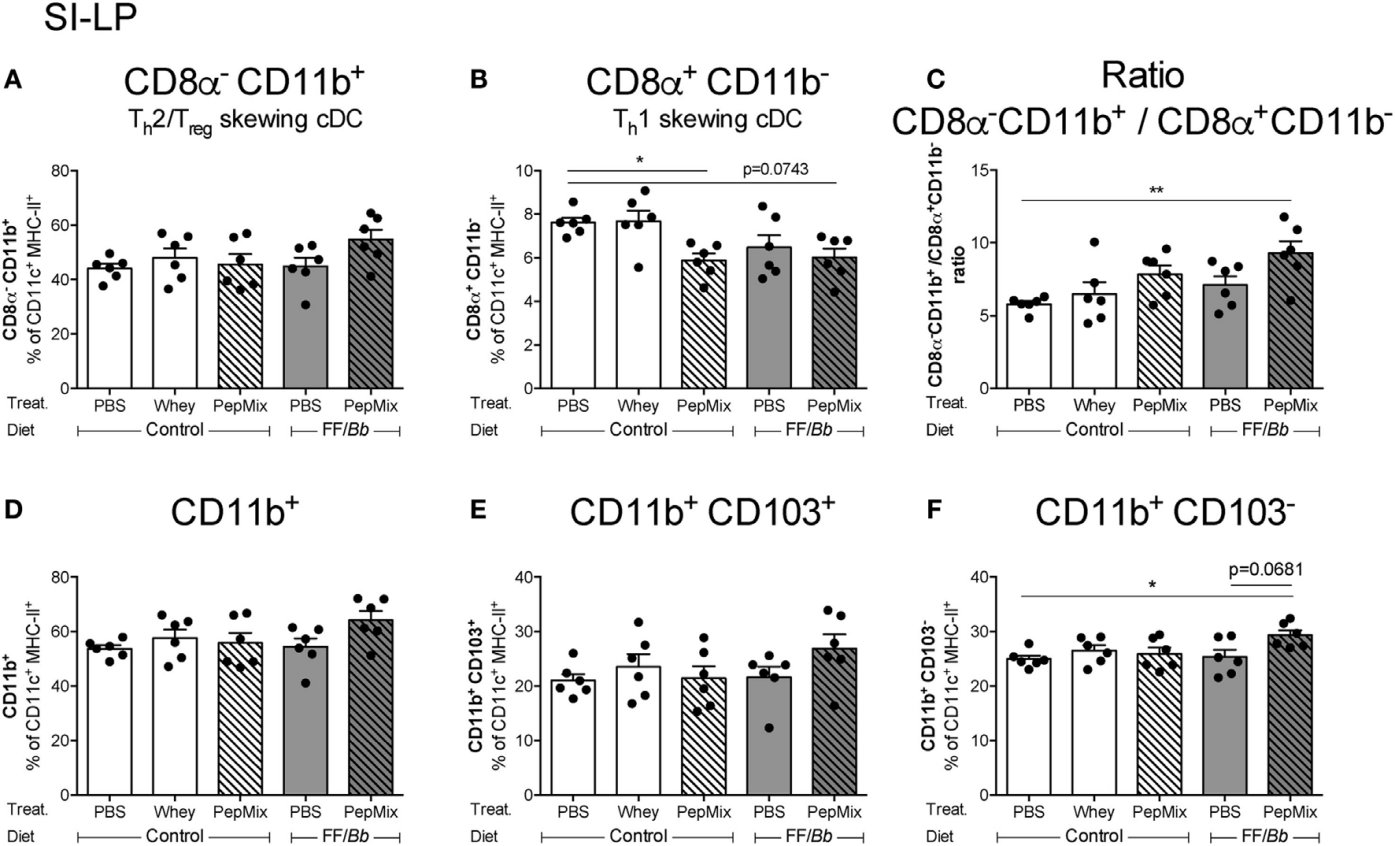
Flow cytometric analyses of dendritic cells (DC) percentages in the small intestine lamina propria (SI-LP) during the oral tolerance phase. SI-LP DC were analyzed based on their expression of CD11c, MHC-II, and lack of F4/80. CD8α^−^CD11b^+^ **(A)** or CD8α^+^CD11b^−^ **(B)** conventional DC (cDC) phenotype and the balance between the two **(C)** were studied. CD11b^+^ myeloid DC **(D)** were further distinguished based on their CD103 expression **(E,F)**. Data are presented as mean ± SEM of *n* = 6/group; *p < 0.05, **p < 0.01 one-way ANOVA followed by Bonferroni’s *post hoc* test for selected groups.

To investigate the impact of the 6-day OT with PepMix and/or FF/*Bb* on the local immune cell phenotype in the gut, flow cytometric analysis of different DC subsets known to reside in the SI-LP (13) was performed. It was observed that PepMix decreased the T_h_1-inducing CD8a^+^CD11b^−^ conventional DC (cDC) (55, 56) and PepMix+FF/*Bb* followed the same tendency (*p* < 0.10) (Figure 9B). On the other hand, the T_h_2/T_regs_-inducing CD8a^−^CD11b^+^ cDC (55, 56) were slightly, but not significantly increased in the PepMix+FF/*Bb* group (Figure 9A). These changes contributed to a significant predominance of CD8a^−^CD11b^+^ over CD8a^+^CD11b^−^ cDC in the SI-LP of the PepMix+FF/*Bb*-exposed mice (Figure 9C). The percentage of CD11b^+^ DC in the SI-LP was not significantly affected in the PepMix+FF/*Bb*-fed mice (Figure 9D), but was significantly increased within the CD103^−^ population (Figure 9F) and not in the CD103^+^ population (Figure 9E). No effect was observed on these DC subsets in the MLN (data not shown).

### Differential Expression of Regulatory Markers in the Small and Large Intestine

**Figure 10:**
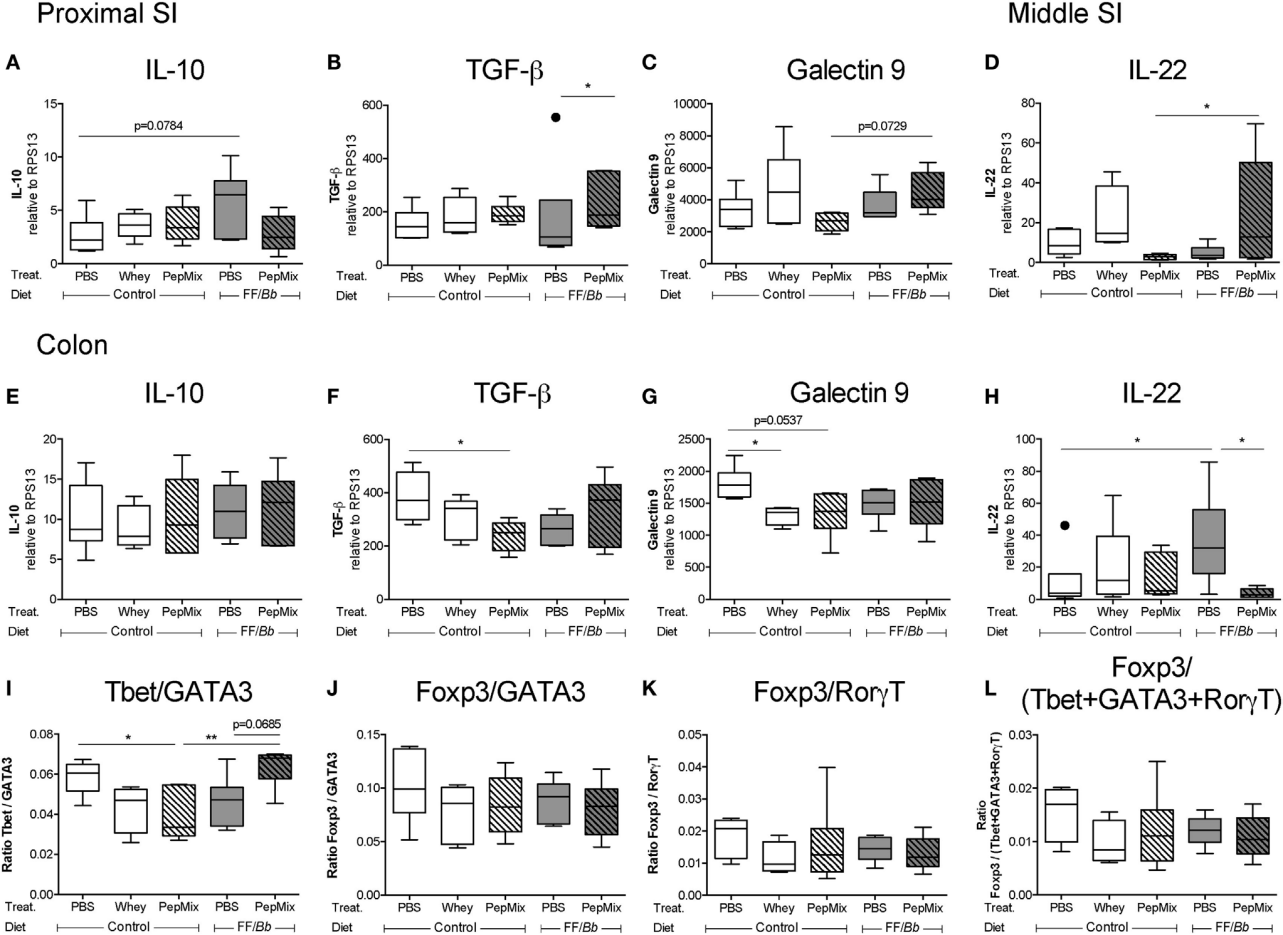
Effect of the peptides and/or dietary synbiotics on the gut during the oral tolerance phase. mRNA expression of potential regulatory markers, such as IL-10, TGF-β, galectin-9, and IL-22, was studied in the proximal small intestine (SI) **(A–C)**, middle SI **(D)**, and in the colon **(E–H)**. The Tbet/GATA3 **(I)**, Foxp3/GATA3 **(J)**, Foxp3/RorγT **(K)**, and Foxp3/(Tbet + GATA3 + RorγT) ratios **(L)** were calculated to represent the balance between the different T cell subsets in the colon. mRNA expression is normalized to the RPS13 housekeeping gene expression. Data are presented as box-and-whisker Tukey plots, *n* = 5–6/group and *n* = 4–6/group for **(K)** and **(L)**; *p < 0.05, **p < 0.01 one-way ANOVA followed by Bonferroni’s *post hoc* test for selected groups after excluding outliers as indicated by Tukey.

The impact of the dietary interventions on the expression of regulatory markers in the proximal and middle SI as well as in the proximal colon was examined (Figure 2B). In the proximal SI, TGF-β mRNA expression was increased in PepMix+FF/*Bb*-fed mice compared to those fed with FF/*Bb* only (Figure 10B). In addition, galectin-9 expression in this group tended to increase compared to mice exposed to PepMix only (Figure 10C). In the middle part of the SI, these markers remained unaffected (data not shown) except for increased IL-22 mRNA levels in the PepMix+FF/*Bb* group compared to PepMix alone (Figure 10D). The expression of regulatory markers in the colon differed from those observed in the SI. Here, galectin-9 expression was suppressed when mice were administered whole whey protein, while the PepMix tended to lower galectin-9 (*p* < 0.10) (Figure 10G) and suppressed TGF-β expression (Figure 10F). On the other hand, FF/*Bb* induced IL-22 expression in the colon, but this was lost in mice that also received PepMix (Figure 10H). Pre-exposure to the FF/*Bb* diet had a tendency (*p* < 0.10) to increase IL-10 expression in the proximal SI compared to PBS control mice (Figure 10A), while IL-10 remained unaffected by the dietary interventions in the colon (Figure 10E).

### PepMix+FF/*Bb* Exposure Preserves the Ratio of T_h_1/T_h_2 Markers in the Colon

The effect of the interventions on transcription factors mRNA expression in the gut was studied. Tbet expression was reduced in the colon of PepMix-exposed mice, whereas this was abrogated in mice that were also fed the FF/*Bb* diet (Figure S2B in Supplementary Material). On the other hand, the expression of GATA3, Foxp3 and RorγT remained unaltered. This resulted in FF/*Bb* restoring the reduced T_h_1 over T_h_2 marker ratio in the PepMix-exposed mice (Figure 10I). No differences were observed on other T cell ratios in the colon (Figures 10J–L) as well as on transcription marker mRNA expression both in the proximal and middle SI (data not shown). These data indicate that the combination of PepMix and FF/*Bb* increases the T_h_1/T_h_2 balance in the colonic tissue compared to PepMix or FF/*Bb* alone and maintains it at the homeostatic level of the PBS control mice.

## Discussion

In the current study, the efficacy of specific short BLG-derived peptides to induce OT during a dietary intervention with FF/*Bb* synbiotics versus a control diet was studied. The preventive effect of the peptides in a whole whey-induced CMA mouse model as well as the possible underlying mechanisms of immunomodulation was studied in both diet groups. Confirming our previous finding with this intervention (35), a significant reduction of the acute allergic skin response to whole whey protein was observed when mice were exposed to PepMix+FF/*Bb* prior to sensitization compared to the PBS-pretreated allergic controls. This result is also in accordance with the findings of Meulenbroek et al. who showed a protective effect of a much higher dose of the same peptide blend alone or in combination with a prebiotic diet (36). Further, we show that PepMix+FF/*Bb* protected mice from developing significantly more severe signs of anaphylaxis compared to the sham-sensitized controls after the i.d. challenge with allergen. In addition, the protective effect of the combined intervention was associated with an increase in activated T cells, among which T_regs_, in the SI-LP. T_regs_ are extensively researched for their involvement in OT, suggesting that the combination of low dose of BLG-derived peptides and FF/*Bb* diet has the potential of facilitating OT development and hence of reducing the allergic response to whole whey protein.

The current study revealed that the T_h_1/T_h_2 balance was increased in the PP of PepMix+FF/*Bb*-fed whey-sensitized mice, as seen both on transcription factor mRNA as well as cytokine mRNA levels. These data are in accordance with previous findings on the preserved T_h_1/T_h_2 balance in the SI-LP of PepMix+FF/*Bb*-pretreated mice (35). Furthermore, reports on the use of dietary synbiotics also suggest their T_h_1-inducing capacities (45), which may partly be contributed to the probiotic bacteria that may increase toll-like receptor activation (TLR) (e.g., TLR9) (57, 58).

Elucidating the working mechanisms of the PepMix+FF/*Bb* preventive exposure in the CMA model might be hindered by the long time-gap between the OT phase and the measurements in the challenge phase. Therefore, the immunomodulatory effects of the interventions were analyzed in mice directly after the OT phase. Changes in the diet, especially the addition of dietary fibers, are known to alter the composition of gut microbiota and also to influence its metabolic activity (50, 59). Microbial products, such as SCFA, are implicated in the maintenance of a tolerogenic mucosal environment (60) and the protection against food allergy development (52). SCFA measurements in the cecal content of PepMix+FF/*Bb*-fed mice revealed increased propionic and butyric acid. Butyrate, but not propionate, is shown to protect mice from developing peanut allergy (52), whereas propionate is found to alleviate airway hypersensitivity in a mouse model of house dust mite-induced asthma (61). SCFA are suggested to support gut homeostasis in various ways, such as affecting immune cells directly, maintaining the balance between pro-inflammatory and anti-inflammatory cells as well as affecting T_regs_ biology (51, 52). Particularly, both propionic and butyric acid are reported to induce *de novo* T_regs_ (51). In line with this, data from our study show that the enhanced concentration of propionic acid was positively correlated with the number of Foxp3^+^ cells in the colonic tissue. Therefore, the role of propionate (and possibly also butyrate) in enhancing Foxp3^+^ cells and in preventing CMA development in PepMix+FF/*Bb*-fed mice needs to be further elucidated.

As PepMix+FF/*Bb* intervention influenced the mRNA expression in the PP, rather than the spleen, of the whey-sensitized mice, the impact of the dietary interventions on the gut was examined during the OT phase. In contrast to preserving the T_h_1/T_h_2 balance in the PP during the challenge phase, PepMix+FF/*Bb* increased the regulatory marker mRNA expression (IL-10 and Foxp3) during the OT phase, suggesting a predominating T_regs_ response in this early period. Although dispensable for tolerance induction, the PP is an important site where early immune responses are shaped (9). Interestingly, IL-22 mRNA expression in this inductive site was enhanced upon PepMix+FF/*Bb* exposure. IL-22 is suggested to have anti-inflammatory effect on non-immune cells (62) and also to improve the intestinal epithelial barrier integrity (63, 64). Stefka et al. further reported that Clostridia-containing microbiota enhanced IL-22, resulting in reduced allergen access to the blood stream and protection against food allergy (63). On the other hand, IL-22 mRNA expression in colon of PepMix+FF/*Bb* mice was differentially affected which might point at a different mechanism of action in the large intestine.

Next to a tolerogenic milieu in the PP, tolerance-related immunomodulation was also observed in the MLN. Oral exposure to PepMix+FF/*Bb* deviated the immune cell response away from the T_h_2, as it induced more Foxp3^+^ cells at the expense of GATA3^+^ cells in the CD4^+^ T_h_ population. Focusing on activated cells co-expressing CD25, it was observed that PepMix+FF/*Bb* reduced CD25^+^Foxp3^−^ T_effs_. Typically in this group, the frequency of CD25^+^ cells tended to decrease, while the proportion of Foxp3^+^ cells within the CD4^+^CD25^+^ subset did increase. This suggests that the PepMix+FF/*Bb* dietary intervention contributes to a more regulatory environment by enhancing the T_regs_ over T_effs_ balance at this inductive site. On the other hand, both activated CD25^+^ T cells and T_regs_ were reduced in the spleen of whey-treated or PepMix+FF/*Bb-*fed mice. This might implicate that in the early period of tolerance induction depletion of activated T cells as well as T_regs_ occurs systemically while *de novo* T_regs_ are induced locally at the intestinal inductive sites, such as the MLN.

After initial generation within the MLN of antigen-fed mice, T_regs_ are reported to undergo a β7-integrin- and CCR9-mediated gut homing followed by a secondary expansion in the SI-LP (65, 66). However, this expansion is suggested to take place over a prolonged period of antigen exposure (67), which would explain the subtle changes in T_regs_ numbers observed in the SI-LP after 6 days of OT. On the other hand, mice exposed to whey sensitization after PepMix±FF/*Bb* OT showed higher T_regs_ frequency in the SI-LP, implying that re-exposure to whole antigen might have contributed to the T_regs_ expansion. Hence, additional studies are needed to explore whether prolonging the OT phase and the exposure to BLG-peptides and synbiotics would result in more T_regs_ and in improved efficiency to prevent CMA.

Further examining the immunomodulatory effects in the gut, it was observed that in the SI-LP there was a shift toward the more T_h_2/T_regs_-inducing CD8a^−^CD11b^+^ cDC subset (55, 56). As we observed decreased T_h_2 cell and increased Foxp3^+^ T_h_ cell numbers in the MLN of PepMix+FF/*Bb-*fed mice, it can be speculated that those DC stimulated the differentiation of naïve T cells to T_regs_ rather than T_h_2 cells. Further, CD11b^+^ myeloid cells tended to increase in the SI-LP during PepMix+FF/*Bb* pretreatment and this elevated pattern remained regardless of their CD103 expression. CD11b^+^ cells are reported to have tolerogenic characteristics in different disease models (68, 69). Even though CD103^+^ DC have been described as the main subtype migrating to the intestinal draining lymph nodes (11), we alike Cerovic et al. have found a proportion of CD11b^+^CD103^−^ DC in the MLN, suggesting that they also migrate (70). Next to the migratory capacity, this CD103^−^ DC subset has been reported to share the ability to efficiently prime T cells and to induce gut tropism as evidenced by their CCR7 expression (70). The same group suggested that the main role of these CD103^−^ DC was to induce T_h_1 and T_h_17 response, but a more recent study reveals that oral mucosa CD103^−^ DC, similar to intestinal CD103^+^ DC, are potent inducers of retinoic acid and Foxp3^+^ T_regs_ (71). Also *in vitro* studies with murine DC report high activity of the vitamin A-converting enzyme, aldehyde dehydrogenase in CD103^−^ cDC (72). Hence, the increased percentage of CD103^−^ DC in the SI-LP might be an essential inducer of Foxp3^+^ T cells in the MLN of PepMix+FF/*Bb*-fed mice and their role in OT induction to whey should be further elucidated.

The immune environment in the gut is known to differ according to the location (73). In line with this, we found that PepMix+FF/*Bb* enhanced the mRNA expression of the regulatory TGF-β in the first part of the SI but not in the colon. This points to a more tolerogenic environment at the location of antigen uptake in the SI. In the colon, on the other hand, the combined BLG-peptides and synbiotics intervention supports the T_h_1/T_h_2 marker balance.

In conclusion, the current study confirms the capacity of a dietary intervention combining low-dose BLG-derived peptides and a diet with FF/*Bb* synbiotics to partially prevent the allergic response to whole whey protein. This coincided with an increased percentage of T_regs_ in the SI-LP and T_h_1/T_h_2 ratio in the PP in the challenge phase. It further suggests that the FF/*Bb* diet might facilitate a more tolerogenic environment during the OT phase by enhancing propionate and butyrate levels in PepMix-pretreated mice. PepMix+FF/*Bb* further stimulates T_regs_ over T_effs_ markers and frequency, as well as the myeloid over conventional DC ratio, and the percentage of CD103^−^ DC. Depending on the location in the intestine, in the PepMix+FF/*Bb* group TGF-β or IL-22 mRNA levels increased which might play role in the facilitation of OT induction by the PepMix. Further refinement of the BLG-peptides and dietary synbiotics approach might contribute to tailoring successful prevention strategies for CMA and possibly also other food allergies.

## Ethics Statement

The animal use was approved by the Animal Ethics Committee of Utrecht University and the Central Commission for Animal use (approval numbers DEC 2013.II.11.116 and AVD108002015262). Animal care and use were conducted in accordance with the guidelines of the Animal Ethics committee of Utrecht University.

## Author Contributions

AK, LK, and LW conceptualized the studies. AK executed the studies, acquired and analyzed the data, and drafted the manuscript; BE and MD participated in the animal study; MD and AP-T participated in the qPCR analysis; MD performed the immunohistochemistry staining. LK, LW, and JG revised the manuscript critically.

## Conflict of Interest Statement

All authors declare no financial or personal conflict of interest in relation to the presented work. LK is employed at Nutricia Research. BE and JG are partly employed at Nutricia Research. AK received funding from Nutricia Research.

## Abbreviations

BLG, β-lactoglobulin; cDC, conventional dendritic cells; CMA, cow’s milk allergy; CMP, cow’s milk protein; CT, cholera toxin; FF/*Bb*, a specific synbiotic mixture of short- and long-chain fructo-oligosaccharides and *Bifidobacterium breve* M-16V; MLN, mesenteric lymph nodes; OT, oral tolerance; PBS, phosphate-buffered saline; PepMix, peptide mixture; PP, Peyer’s patches; SCFA, short-chain fatty acids; SI, small intestine; SI-LP, small intestine lamina propria; T_h_, T helper cells; T_regs_, regulatory T cells; T_effs_, effector T cells.
