# Supplementary material for: Dietary Intervention with β-Lactoglobulin-Derived Peptides and a Specific Mixture of Fructo-Oligosaccharides and Bifidobacterium breve M-16V Facilitates the Prevention of Whey-Induced Allergy in Mice by Supporting a Tolerance-Prone Immune Environment
Source: Front Immunol. 2017 Oct 23;8:1303. doi: 10.3389/fimmu.2017.01303 (PMC5662887; doi:10.3389/fimmu.2017.01303)
Supplement: Supplementary file 1 [file Image_1.PDF]

Supplementary Material

Dietary intervention with β-lactoglobulin-derived peptides and a specific mixture of fructo-oligosaccharides and *Bifidobacterium breve* M-16V facilitates the prevention of whey-induced allergy in mice by supporting a tolerance-prone immune environment

Atanaska I. Kostadinova, Alba Pablos-Tanarro, Mara A.P. Diks, Betty C.A.M. van Esch, Johan Garssen, Léon M.J. Knippels, Linette E.M. Willemsen*

*** Correspondence:** Linette E.M. Willemsen, Division of Pharmacology, Utrecht Institute for Pharmaceutical Sciences, Utrecht University, PO Box 80195, 3805TD Utrecht, The Netherlands
l.e.m.willemsen@uu.nl

1 Supplementary Figures and Tables

1.1 Supplementary Figures

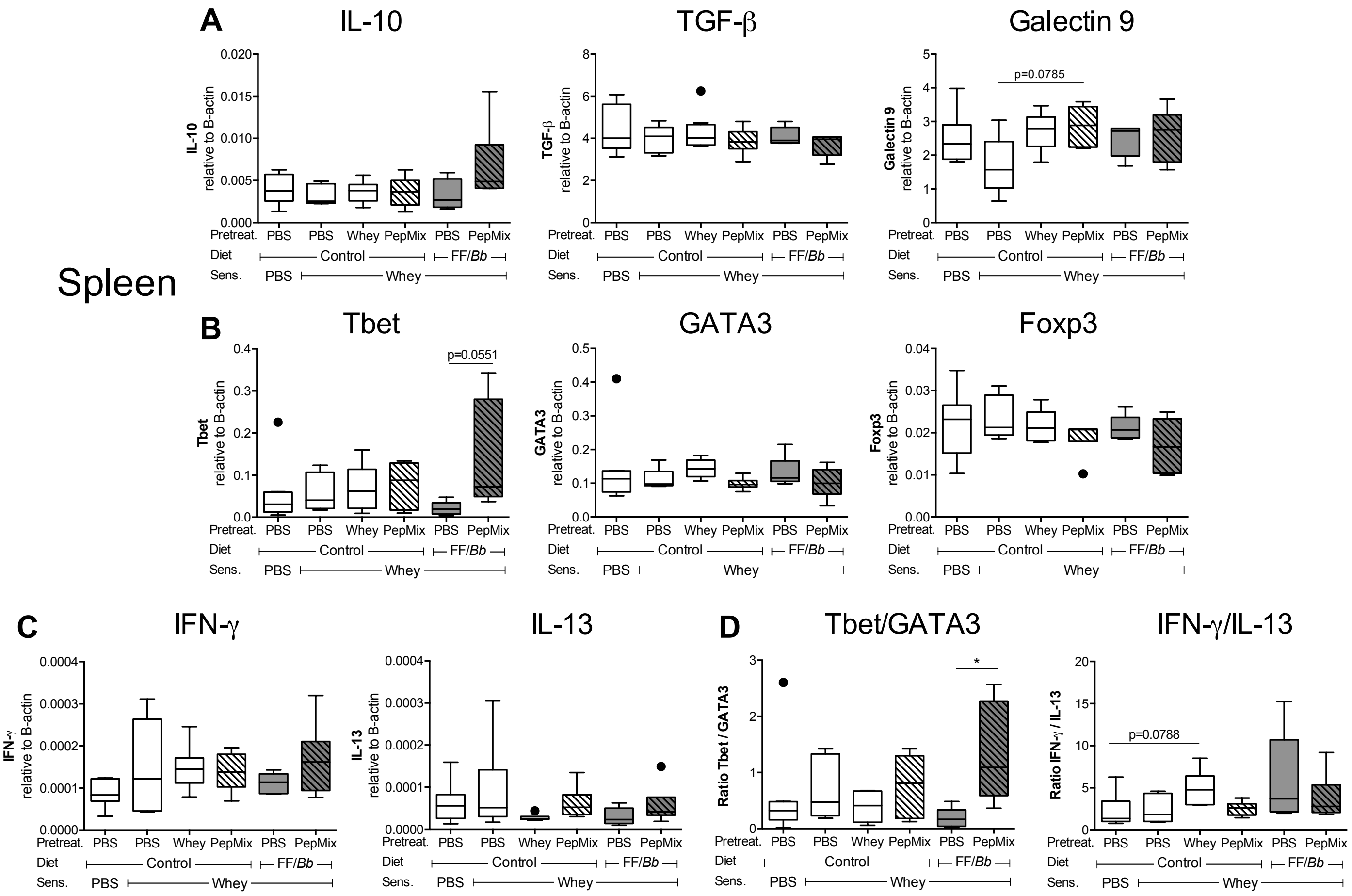

Figure S1 mRNA expression in the spleen after the challenge phase. Spleens were collected 18 h after the oral challenge and used for mRNA isolation and cDNA synthesis. mRNA expressions of the regulatory IL-10, TGF-β and galectin-9 markers **(A)**, Tbet, GATA3, and Foxp3 transcription factors **(B)** and T_h1-associated IFN-γ and T_h2-associated IL-13 **(C)** were measured using a real-time qPCR. The ratios of Tbet/GATA3 and IFN-γ/IL-13 **(D)** were calculated to represent the T_h1/T_h2 balance. mRNA expression is normalized to the B-actin housekeeping gene expression. Data are presented as box-and-whisker Tukey plots, n=4-8/group for IL-10 and n=5-8/group for all others; * $p<0.05$ one-way ANOVA followed by Bonferroni's *post hoc* test for selected groups.

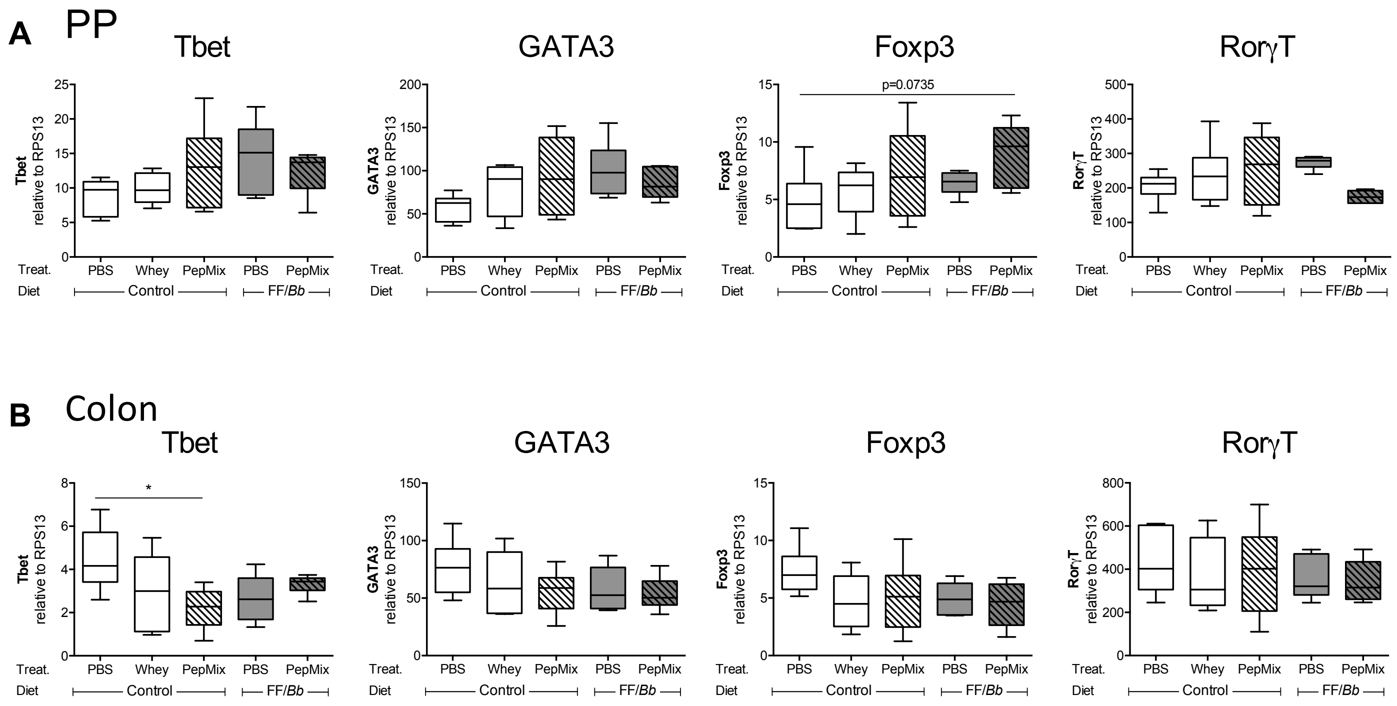

Figure S2 Impact of the dietary interventions on the transcription factors in the PP and colon during the OT phase. PP **(A)** and colon **(B)** were collected 4 h after the last administration of the OT phase in order to study the mRNA expression of Tbet, GATA3, Foxp3 and RorγT transcription factors. mRNA expression is normalized to the RPS13 housekeeping gene. Data are presented as box-and-whisker Tukey plots, n=5-6/group and analyzed with one-way ANOVA followed by Bonferroni's *post hoc* test for selected groups.

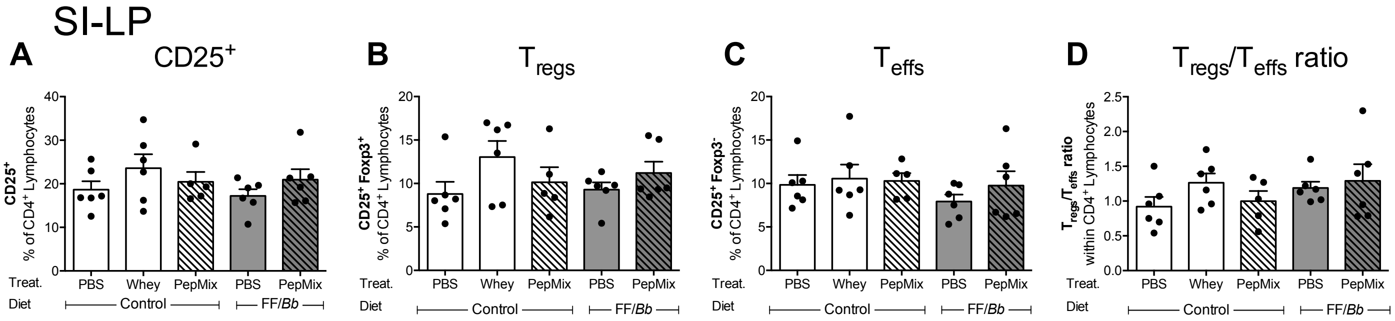

Figure S3 Flow cytometric analysis of T cells in the small intestine during OT phase. SI-LP were collected 4 h after the last oral administration of the OT phase. Percentages of $CD25^{+}$ T cells (A), $CD25^{+}Foxp3^{+}$ T_{regs} (B) and $CD25^{+}Foxp3^{-}$ T_{effs} (C) were determined within the $CD4^{+}$ lymphocyte pool and the balance between T_{regs} and T_{effs} (D) was investigated. Data are presented as mean ± SEM n=5-6/group.
